# Consensus on the treatment of vitiligo – Brazilian Society of Dermatology^☆^^☆☆^

**DOI:** 10.1016/j.abd.2020.05.007

**Published:** 2020-10-10

**Authors:** Gerson Dellatorre, Daniela Alves Pereira Antelo, Roberta Buense Bedrikow, Tania Ferreira Cestari, Ivonise Follador, Daniel Gontijo Ramos, Caio Cesar Silva de Castro

**Affiliations:** a Department of Dermatology, Hospital Santa Casa de Misericórdia de Curitiba, Curitiba, PR, Brazil; b Department of Dermatology, Universidade do Estado do Rio de Janeiro, Rio de Janeiro, RJ, Brazil; c Department of Dermatology, Santa Casa de Misericórdia de São Paulo, São Paulo, SP, Brazil; d Department of Dermatology, Hospital de Clínicas de Porto Alegre, Porto Alegre, RS, Brazil; e Private Clinic, Salvador, BA, Brazil; f Department of Dermatology, Santa Casa de Misericórdia de Belo Horizonte, Belo Horizonte, MG, Brazil; g Department of Dermatology, Faculty of Medicine, Pontifícia Universidade Católica do Paraná, Curitiba, PR, Brazil

**Keywords:** Clinical protocols, Combined modality therapy, Consensus, Treatment outcome, Vitiligo

## Abstract

**Background:**

Vitiligo is a muco-cutaneous, autoimmune, localized, or disseminated disease, which manifests through hypochromic or achromic macules, with loss in quality of life. The prevalence of vitiligo in Brazil was determined to be 0.54%. There is no on-label medication for its treatment. To date, no Brazilian consensus on the treatment of vitiligo had been written.

**Objectives:**

The objective of this group of Brazilian dermatologists with experience in the treatment of this disease was to reach a consensus on the clinical and surgical treatment of vitiligo, based on articles with the best scientific evidence.

**Methods:**

Seven dermatologists were invited, and each was assigned two treatment modalities to review. Each treatment (topical, systemic, and phototherapy) was reviewed by three experts. Two experts reviewed the surgical treatment. Subsequently, the coordinator compiled the different versions and drafted a text about each type of treatment. The new version was returned to all experts, who expressed their opinions and made suggestions for clarity. The final text was written by the coordinator and sent to all participants to prepare the final consensus.

**Results/Conclusion:**

The experts defined the following as standard treatments of vitiligo: the use of topical corticosteroids and calcineurin inhibitors for localized and unstable cases; corticosteroid minipulse in progressive generalized vitiligo; narrowband UVB phototherapy for extensive forms of the disease. Surgical modalities should be indicated for segmental and stable generalized vitiligo. Topical and systemic anti-JAK drugs are being tested, with promising results.

## Introduction

Vitiligo is a muco-cutaneous disease, triggered by autoimmunity against melanocytes, which is manifested by hypochromic or achromic macules and patches. Lesions can be localized or disseminated and have a very negative impact on quality of life. The disease can appear with one or a few macules grouped in a non-segmental way (focal vitiligo) or even reach the entire tegument (universal vitiligo).

Vitiligo is classified into two groups: non-segmental (NSV), which comprises the focal, mucosal, acrofacial, common, and universal types, and the segmental group, which includes only the segmental type. This type usually affects only one hemibody and, in most cases, presents a slower response to non-surgical treatment than the NSV type. In segmental vitiligo, autoimmunity against melanocytes is also present, but it is believed that an autoimmune attack occurs only against a certain area of cutaneous mosaicism.^1^ Vitiligo can also be chemically induced, mainly by phenolic derivatives.^2^

The prevalence of vitiligo in Brazil was determined to be 0.54%; the mean age of onset of vitiligo in Brazilian patients for segmental and common types was determined to be 13 and 22 years old, respectively.^3^, ^4^

The genetic influence on the onset of vitiligo has already been well determined; a recent genetic study identified an increased polygenic load of risk alleles identified by genome-wide association studies (GWAS) in patients from families with multiplex cases (with family history) when compared with simplex patients (no family history).^5^ The lesions are caused by effector autoreactive CD8^+^ T lymphocytes in the initial phase, and in the stable phase they are determined by recirculating CD8^+^ T lymphocytes, both responsible for destroying melanocytes through the cytotoxic action of the released granzymes and perforin.^6^

According to the definition of the Vitiligo Global Consensus, analysis of vitiligo stability or instability should include a combination of history, series of photographs, and clinical scores such as VASI or VETF; vitiligo is considered stable if no new lesions, enlargement of old macules, Köbner phenomenon, or perifollicular (confetti) depigmentation is observed.^7^, ^8^

There are no specific treatments for vitiligo – for instance, no medication has been developed and approved specifically for its treatment by the United States Food and Drug Administration (FDA). This article, written by seven Brazilian dermatologists, aimed to reach a consensus on clinical and surgical treatment of vitiligo based on a review of the literature with the best scientific evidence available to date.

## Methods

Seven dermatologists were invited, and each was assigned two treatment modalities to review. Three experts individually reviewed the literature and wrote the guidelines for topical, systemic, and phototherapy treatment. Two experts reviewed the surgical modality. Subsequently, the coordinator compiled the different versions and drafted a text about each type of treatment. The new version was returned to all experts, who expressed their opinions and made suggestions for clarity. The final text was written by the coordinator and sent to all participants to prepare the final consensus.

## Topical therapies

The goal of vitiligo treatment is to halt the progression of the disease, stimulate pigmentation, and finally, maintain repigmentation, thus circumventing the psychosocial impact caused by this dermatosis.

For small areas or when other alternatives are not available, the treatment of choice is topical. Topical treatment combined with phototherapy is indicated in therapeutic failure or when more than 5% to 10% of the body surface is affected.^9^

Although other clinical and surgical treatments are available, topical treatment with corticosteroids and calcineurin inhibitors has a prominent role in the therapeutic arsenal of vitiligo.

### Topical corticosteroids

Topical corticosteroids in monotherapy are the first line in the treatment of localized unstable vitiligo, and can also be used in combination with phototherapy in generalized lesions. Recent and facial lesions present the best response.^10^

The mechanism of action of corticosteroids in vitiligo has not yet been fully elucidated, but an immunohistochemical study with mometasone in vitiligo induced by Köbner phenomenon observed a significant decrease in the infiltration of CD4^+^ and CD8^+^ T lymphocytes.^11^

In 1988, a meta-analysis concluded that the use of class III and IV corticosteroids resulted in over 75% of repigmentation when compared with placebo.^12^ Other studies have shown that 0.05% clobetasol propionate is especially effective on the face and neck.^13^, ^14^

A prospective, randomized study was conducted to estimate the safe dose of high-potency corticosteroids in vitiligo. The authors demonstrated that weekly use of 50 g (or less) of 0.05% clobetasol propionate cream for 12 weeks is safe, with no evidence of adrenal insufficiency, although local effects may occur.^15^ To minimize the risk of side effects, topical corticosteroids should be limited to small areas, avoiding prolonged use on the face and flexures.

It is recommended that, after eight weeks of continuous topical corticosteroid application, another topical therapy (rotational therapy) is introduced.^12^, ^16^ In practice, these therapeutic schemes appear to minimize side effects, although evidence-based studies to confirm this information are still lacking.^9^ If no repigmentation is observed after three months of application, this treatment should be interrupted. Six of the present reviewers voted that their use is safer when quantified in grams; and one was in favor of the product being used on less than 10% of the body surface.

### Calcineurin inhibitors

Calcineurin inhibitors have been shown to be effective and safe in the treatment of vitiligo in both children and adults, although their use is considered off-label by the Brazilian National Health Surveillance Agency (ANVISA). A recent meta-analysis comparing the use of calcineurin inhibitors *vs*. topical corticosteroids of medium or high potency showed that calcineurin inhibitors are not inferior to topical corticosteroids for the treatment of vitiligo, especially in children.^17^

Tacrolimus and pimecrolimus are the main calcineurin inhibiting agents. Tacrolimus is a macrolide product of the bacterium *Streptomyces tsukubaensis*, which has immunomodulatory properties and acts by selective inhibition of the intracellular protein calcineurin. It is not known exactly what is the mechanism of action of tacrolimus in vitiligo, but in an immunohistochemical study with 0.1% tacrolimus in vitiligo induced by the Köbner phenomenon, a significant decrease was observed in the infiltration of CD4^+^ and CD8^+^ T lymphocytes.^11^

The effectiveness of calcineurin inhibitors in vitiligo has been verified in a series of publications since 2002.^18^, ^19^ The best results are observed on the face and in photoexposed areas.^20^, ^21^ The efficacy of tacrolimus was compared with that of 0.05% clobetasol, a high-potency corticosteroid.^22^ Although pimecrolimus has a more acceptable presentation (cream), the vast majority of the studies address tacrolimus.^23^

In a prospective, controlled study, 0.1% tacrolimus ointment used twice per day for one year led to the repigmentation of facial lesions in 81% of patients; in turn, repigmentation was minimal on the limbs. Its advantage is that it is well tolerated by children and adults, and can be used for prolonged periods of time without the undesirable effects of corticosteroid therapy, such as atrophy and telangiectasis.^24^

Calcineurin inhibitors can be prescribed twice per week as a maintenance therapy, after repigmentation of lesions treated with phototherapy.^25^

There are two presentations of tacrolimus: 0.03% (ointment; approved for use in children between 2 and 15 years) and 0.1%. In 2013, a European consensus proposed that calcineurin inhibitors applied twice daily should be the first choice to treat head and neck lesions.^26^

Topical calcineurin inhibitors are safe for continuous use, for short or prolonged periods, or intermittently.^16^ Their side effects include burning, irritation, ardency, and pruritus, which tend to subside over time.

The FDA included a black box warning on the medication package as a preventive measure, based on the theoretical risk of lymphoma and non-melanoma skin cancer, based on studies in animal models and the use of a systemic calcineurin inhibitor.^16^ An analysis of data from more than 20,000 clinical trials has found that, to date, there is no evidence to suggest an increased risk of lymphoma or non-melanoma skin cancer in children or adults.^16^, ^27^, ^28^

There is concern regarding the induction of skin cancer when combining the topical use of calcineurin inhibitors with phototherapy. However, Tran et al. showed that 0.1% tacrolimus and 1% pimecrolimus can prevent DNA photodamage in mice, by reducing the production of thymine dimers triggered by UVB radiation.^29^ Among the present authors, there was no consensus on how long to use topical tacrolimus. Likewise, no consensus was reached regarding maximum body area, due to lack of sufficient data in the literature.

### Other topics

Calcipotriol, pseudocatalase, and khellin were mentioned by some of the present authors, but no consensus was reached regarding the indication for its use.

No consensus was reached among the panelists regarding indication of depigmentation with monobenzyl ether of hydroquinone in very extensive vitiligo, for the following reasons: it is not a treatment for vitiligo, but for normal skin; it is not sold or approved in Brazil; and there is a potential non-reversibility of skin color after use. The experts also unanimously advised against the use of lasers or phenol peels for permanent depigmentation, which are controversial, due to the possible irreversibility of the clinical picture and the advance of new drugs that are about to be launched.

Three new topical medications from the Janus kinase inhibitor (anti-JAK) class are being tested: topical ruxolitinib, in Phase III, and two systemic drugs inhibiting the JAK3/TEC and TYK2/JAK1 pathways, in Phase II.

https://clinicaltrials.gov/ct2/show/NCT04057573?cond=vitiligo&draw=3&rank=18; https://clinicaltrials.gov/ct2/show/NCT04052425?cond=vitiligo&draw=3&rank=19; https://clinicaltrials.gov/ct2/show/NCT04103060?cond=vitiligo&draw=4&rank=22.

## Systemic therapies

### Corticosteroids

Oral corticosteroids have a broad immunosuppressive spectrum and aim to contain the progression of lesions in patients with active disease. The primary objective is, therefore, to stop the onset of new lesions and, secondarily, to induce repigmentation. The mode of action of corticosteroids in vitiligo has not yet been elucidated. A single article demonstrated a decrease in serum ICAM1 after using oral minipulse therapy (OMP) for vitiligo.^30^ Its systemic use, most often prescribed in minipulses, has been evaluated in some studies, most of them open.^31^

The most widely used form of systemic corticosteroid therapy is the administration of oral minipulses of corticosteroids, usually of betamethasone or dexamethasone, in supra-pharmacological doses and intermittently, in order to reduce the side effects associated with daily administration. Doses range from 2.5 to 10 mg dexamethasone for two consecutive days/week for three to six months. Betamethasone is usually prescribed in this same dosage schedule, in doses between 5 to 7.5 mg. In theory, any oral corticosteroids could be used, respecting the equivalence. The number of patients in whom an interruption in the progression of vitiligo was observed varies little between studies, from 88% to 91.8%. In three studies, the onset of some degree of repigmentation was observed in 28% to 100% of patients.^32^, ^33^, ^34^ Studies comparing treatment with OMP with daily use of corticosteroids observed a lower incidence of systemic and skin adverse effects in the former.^16^ The OMP regimen can be combined with phototherapy in patients with progressive vitiligo, although controlled studies with long-term follow-up are still required.^35^, ^36^

The side effects of using OMP are generally mild (described in 69% of patients in the study by Radakovic-Fijan et al.). The following were mentioned: weight gain, insomnia, acne, increased appetite, agitation, hypertrichosis, headache, and lethargy. However, despite the frequent occurrence of adverse effects, they do not usually compromise treatment adherence and do not suppress the production of endogenous cortisol.^32^, ^33^

A consensus was reached regarding the recommendation of the use of OMP, the most used being dexamethasone and betamethasone; daily use of oral corticosteroids was not contraindicated by the present authors. The most frequently mentioned reason for not preferring continuous use was the smaller number of studies when compared with OMP, primarily because there is no standard of total treatment duration and dose reduction scheme.

### Methotrexate

It is indicated to stop the progression of vitiligo whenever oral corticosteroids are contraindicated or to avoid their long-term risks. Studies show results in terms of halting disease progression. The mode of action of methotrexate in vitiligo is not yet known.

In a randomized comparative study, methotrexate 10 mg/week was compared with OMP (2.5 mg dexamethasone/day on two consecutive days per week) in patients with progressive vitiligo (*n* = 52 patients), for 24 weeks. In both groups, the same percentage of reduction in disease activity and in unresponsive patients was observed (6/25 patients in the MTX group and 7/25 in the dexamethasone OMP group). Most studies suggest low doses, around 10–15 mg/week.^37^, ^38^

The authors did not reach a consensus regarding the use of methotrexate, and do not recommend it, mainly due to the lack of a defined adequate dose, and the lack of studies with larger populations. Some of the experts suggest that the use of methotrexate has some scientific basis and spares the use of oral corticosteroids.

### Azathioprine

A single randomized study was conducted comparing the effect of azathioprine, 50 mg twice daily, with 5 mg betamethasone OMP on two consecutive days. In the second month of treatment, vitiligo was stabilized in 19 of 23 patients treated with OMP and in four of 22 treated with azathioprine.^39^ None of the authors indicated the use of this medication.

### Oral antioxidants

Oxidative stress and free radicals play an important role in the pathogenesis of vitiligo.^40^

A systematic review with an attempt at meta-analysis found that the trials with antioxidants had a small number of patients and a wide variety of compounds and protocols, which hindered the comparison of the studies. Thus, the effectiveness of antioxidants alone cannot be confirmed. The best evidence was demonstrated in three studies with *Ginkgo biloba* as monotherapy, demonstrating clinical benefits or decreased inflammation.^40^

The action of antioxidants associated with phototherapy was analyzed in a systematic review and meta-analysis of four studies (PUVA: 1 study and UVB-FE: 3 studies) involving 91 patients who met the inclusion criteria; in two studies, *Polypodium leucotomos* was used, and in another, *Ginkgo biloba*. The association was shown to be more effective than phototherapy alone (relative risk = 1.87; 95% confidence interval: 1.10–3.17). Sensitivity analysis revealed that the result was robust and not dependent on any individual study.^41^

The present panel of authors highlighted evidence of the use of antioxidants in vitiligo, mainly associated with phototherapy, but there was no consensus for the use of these medications, primarily due to the lack of prospective studies with larger populations.

## Modalities of phototherapy and laser

Exposure to the sun, associated with the ingestion or topical application of plant extracts, has been used as a therapeutic agent for vitiligo for over 3000 years. Over the decades, the use of phototherapy has been consolidated through scientific studies, evidence-based medicine, and clarification of the mechanisms of action of ultraviolet radiation (UVR) on human beings.^42^

Phototherapy promotes skin repigmentation through various mechanisms, including the differentiation and migration of melanocytes from the hair follicle and the formation and transfer of melanosomes to keratinocytes, in addition to its immunosuppressive effect.^43^

The immunosuppressive effects of narrowband ultraviolet B radiation (NB-UVB) are already well characterized: apoptosis of T cells, downregulation of inflammatory cytokines, upregulation of interleukin 10 (IL10), and depletion of Langerhans cells, with reduced presentation of antigens. There is also the stimulation of tyrosinase with increased melanin synthesis.^42^

The most used forms of phototherapy for vitiligo include NB-UVB, excimer laser (ExLs), excimer lamp (ExLp), and ultraviolet A with administration of oral and topical psoralens (PUVA). Many of these modalities have shown better results when associated with systemic or topical treatments.

### Phototherapy

Regarding the indication of phototherapy in relation to disease activity, most studies do not specify whether or not the disease is active, or whether or not it is stable; phototherapy is used in both forms.

In unstable vitiligo, in which lesions are progressing, it is recommended to combine phototherapy with systemic corticosteroid therapy, preferably in the form of OMP, as described in the specific part of systemic treatment, as well as the association with oral antioxidants.

Topical medications, such as calcineurin inhibitors (tacrolimus and pimecrolimus) and corticosteroids, can be used in conjunction with phototherapy. The results are variable. Some studies suggest that the association of these topical medications with phototherapy increases the response; however, other studies failed to confirm this action.^42^, ^43^, ^44^, ^45^, ^46^

Although the use of a light source and tacrolimus is contraindicated in the package insert, due to the evidence presented, the group agreed that tacrolimus can be used in conjunction with phototherapy, except immediately before irradiation.

In stable vitiligo, phototherapy acts on repigmentation and can also be associated with the aforementioned oral antioxidants and topical medications, with some studies suggesting a better response with their association.^16^, ^43^, ^47^

As for the clinical form and location of the lesions, rapidly progressive vitiligo accompanied by early poliosis and SV tend to be unresponsive to phototherapy;^48^ however, a recent study has shown that patients with recent SV respond better to phototherapy than those with long-term illness.^49^ It is well established that generalized vitiligo (or NSV) responds better than SV.^50^

The hairy areas, and especially the face and neck areas, show better response rates to phototherapy, followed by the trunk, limbs, hands and feet.^51^, ^52^, ^53^ Likewise, patients with phototypes above III usually have a better prognosis with phototherapy.

Previous history of diseases influenced by light and photosensitivity are potential contraindications to phototherapy, as well as the use of medications at risk of photosensitization.^54^ In addition, it is contraindicated in patients with a history of keratinocytic skin cancer and melanoma.

For the treatment of extensive, generalized vitiligo lesions, phototherapy with NB-UVB or PUVA in cabins is indicated. In localized vitiligo, it is recommended to use emitters of NB-UVB or local UVA, aimed only at the lesions. ExLs and ExLp are also recommended for this type of localized lesion.^55^, ^56^

In children, it is necessary to ensure that there is a correct indication for this therapeutic modality. The level of evidence for the use of NB-UVB in children is 4, but there is no consensus on the age of onset of topical or cabin NB-UVB. Topical PUVA can be performed on children and, for oral PUVA use, the British Photodermatology Group has established two criteria: a broader criterion, that states that it should be used only in those above 10 years, and another criterion that states PUVA should be used in those above 16 years.^57^, ^58^, ^59^

Regarding pregnancy, there is no evidence of a decrease in serum folic acid after exposure to UVA. Studies in patients undergoing NB-UVB have shown different results, potentially explained by the dose-dependent degradation of folate. An accumulated exposure of >40 J/cm^2^ and >2 J/cm^2^ per treatment session was associated with a 19–27% decrease in serum folic acid levels, while lower doses did not affect these levels.^60^ There is no evidence that PUVA is teratogenic, and it should be considered as a second option in phototherapy, due to the need to use psoralen.^59^ This group reached the consensus that pregnant women can be treated with NB-UVB.^61^

Regarding the radiation modality, the role of phototherapy in the treatment of vitiligo has been well established in the last decades and, from the 1990s onwards, studies have been showing advantages of phototherapy with NB-UVB in relation to PUVA phototherapy.^62^, ^63^ While some studies failed to demonstrate clinical superiority, they found similar responses and operational advantages.^64^ Several studies have demonstrated greater effectiveness of NB-UVB compared to PUVA, in addition to its ease of use, without the need for psoralen, which has made phototherapy with NB-UVB the first choice for the treatment of vitiligo.^52^ The NB-UVB phototherapy protocols will not be detailed here and should be the subject of another specific consensus.

### Excimer laser

ExLs is characterized by a wavelength of 308 nm (generated using xenon and chlorine gases). Several studies demonstrate its applicability for the treatment of vitiligo and it is considered to have a similar result to NB-UVB and, in some studies, even higher, with a faster response.^65^, ^66^ Due to its smaller emission area, ExLs reaches smaller areas and regions more difficult to access with standard emitters. However, this form of treatment is inappropriate for patients with more extensive affected areas or for multiple regions. Furthermore, the cost is higher than that of other phototherapy devices.

### Excimer lamp

The monochromatic excimer light (ExLp) also emits light with a wavelength of 308 nm, with good results in inducing repigmentation. These lamps have a larger treatment field than ExLs, making it possible to treat larger areas in a shorter time. In addition, its costs are much lower than laser devices.^67^

Several studies have compared the therapeutic efficacy of ExLs, ExLp, and NB-UVB in vitiligo.^68^, ^69^, ^70^ Recent meta-analyses have shown that the excimer treatment is more effective than NB-UVB, with a faster effect and a higher degree of repigmentation.^71^, ^72^ However, other authors question this greater effectiveness.^72^

As for photocarcinogenesis, the risk of developing skin cancers in patients with vitiligo in the affected areas is rare, regardless of the type of phototherapy.^73^

In vitiligo, PUVA treatment does not appear to be associated with the risk of non-melanoma skin cancer.^74^, ^75^ There are no consolidated studies on the risk of photocarcinogenesis in NB-UVB phototherapy for vitiligo.^73^

There is no concrete evidence that NB-UVB presents a greater risk of malignancy in the genital areas; nonetheless, it is recommended that no exposure be made.^76^

Treatments with ExLs or ExLp showed good results in lesions of the genital area, especially in men.^53^ However, the group agreed that the genital area should not be exposed to phototherapy, due to the previously reported risk of squamous cell carcinomas in patients undergoing phototherapy without adequate protection.^77^

As for the number of therapeutic sessions, a recent study identified patterns of response to treatment with NB-UVB. After 24 sessions, the mean global improvement was 21.5% (579 patients), and these were divided into response time patterns: after 96 sessions, very fast responders presented 88% repigmentation; fast responders, 74.8%; medium responders, 58.4%; slow responders, 38.8%; and non-responders, 35.2%.

The maximum number of sessions recommended for PUVA treatment is 200, but to date there is no global consensus on the maximum number of NB-UVB sessions.^59^, ^73^ Likewise, the present authors did not reach a consensus on the maximum number of sessions for NB-UVB for vitiligo.

To assess the effectiveness of phototherapy, a period of at least six months (approximately 48 sessions) of treatment is required.^52^ In vitiligo, in order to obtain the maximum response with phototherapy, a long treatment time is recommended, of at least one year (on average 96 sessions).^52^

In one year (approximately 96 sessions) of phototherapy, a better response is observed on the face and neck, followed by the trunk, whereas the limbs (hands and feet) show little repigmentation of the lesions.^52^

In conclusion, phototherapy is an excellent therapeutic tool for the treatment of vitiligo, if the correct indications, periodic monitoring, protocols, and individualized management are adopted.

## Surgical treatments

Surgical treatment of vitiligo is indicated for stable cases that are refractory to clinical treatments.^78^, ^79^ Although there is a tendency for a better response in cases of SV, any stable clinical type of the disease can be treated, although specific areas (such as lips, glans, and distal phalanges) present a much lower response to these treatments.^80^, ^81^ If the patient has more than 10% of compromised body surface, even in case of stable and late evolution, surgery is not recommended unless melanocyte culture is available. However, in specific localized cases with more than 10% of affected area, surgical techniques can be used in more than one step. Cases of long-lasting SV (more than 12 months) tend to have less response to clinical treatments.^49^ Therefore, in these cases, surgical treatment can be used as the first therapeutic option.^9^

Vitiligo stability is defined as the absence of new lesions or absence of an increase in old lesions; the indicators of disease instability are Köbner phenomenon, trichromic macules, and confetti depigmentation.^82^ Although there are no definitive parameters on the duration of disease stability required before a surgical approach, it is a consensus among most authors that the mean stability time is 12 months.^83^, ^84^

Stability can be confirmed by the patient, by photographic comparison or validated systems such as Vitiligo Area Scoring Index (VASI), Vitiligo European Task Force Assessment (VETF), and Vitiligo Disease Activity (VIDA).^9^ In cases of difficulty in defining stability, the mini-grafting test must be performed.^78^, ^85^, ^86^

Patients should be evaluated for a history of keloids, coagulopathies, infections, or other contraindications for surgery.^87^ Moreover, they need to be aware of the need for occlusive dressing in the treated area for a period ranging from seven to 14 days. These accelerate the healing of the dermabrased areas, prevent bacterial contamination, and maintain the transplanted tissues or cells in the recipient areas.^88^

The choice of the surgical modality to be used depends on some parameters, such as the size of the lesion, the anatomical area to be treated, and the surgeon's experience.^78^ Cell suspension techniques, although simplified in recent decades, demand a greater learning curve due to the delicate acquisition of tissue from the recipient area, laboratory handling of the tissue, and preparation of the recipient area.^89^

Surgical modalities can be classified into tissue and cellular techniques, according to the type of graft to be transplanted. Among them, tissue melanocyte transplantation, mini-grafting, suction blister epidermal grafting, partial skin grafts, and epidermal curettage are noteworthy. In turn, cellular techniques include melanocyte-keratinocyte transplant procedure (MKTP), suspension of epidermal cells of the follicular external sheath, and suspension of cultured cells.^79^, ^90^

The therapeutic response to the aforementioned techniques ranges from good to excellent (50–100% repigmentation) in more than 65% of patients treated with a single procedure, varying according to the technique used, anatomical area treated (facial areas present better response than acral areas), time of stability, and type of vitiligo (SV responds better than focal, which in turn responds better than generalized).^87^, ^91^, ^92^, ^93^, ^94^, ^95^, ^96^, ^97^, ^98^, ^99^ Furthermore, cellular modalities have the advantage of using a 1:10 ratio between donor area and recipient area, while tissue techniques usually demand a ratio of up to 1:1.^9^, ^79^

Tissue transplantation, such as mini-grafting, has the advantage of being easier, without the need for laboratory equipment, and faster, as long as the area to be treated is small. Modifications of the technique, using motorized punch devices, have allowed the use of this technique in larger areas.^98^, ^100^ Cellular cell transplant modalities, such as MKTP have the advantage of treating larger areas using a reduced donor area, with a lower incidence of side effects than those observed in tissue techniques, making it one of the gold standard techniques for the surgical treatment of vitiligo.^9^, ^79^, ^91^

In turn, techniques that involve the cultivation of melanocytes, in spite of their high efficacy, require a complex laboratory structure and a specialized team and, therefore, have a high cost.^79^ Although there are no reports of post-transplant malignancy outcomes, there are theoretical questions about the long-term behavior of cultured melanocytes, and this technique should initially be reserved for experimental studies with the approval of a local ethics council until studies with long follow-up periods are carried out.^101^

Before the procedures, it is recommended to obtain a signed consent form from all patients, since surgical techniques can culminate in unwanted effects such as Köebner’s phenomenon, unsightly scars (both in the recipient and donor areas), infections, and allergic reactions.^78^, ^93^ Especially in the case of mini-grafting, the cobblestone appearance in the recipient area can occur in about 18–33% of treated patients (lower incidence when using smaller diameter grafts), in addition to an aspect of heterogeneous repigmentation in 24–43%.^98^, ^99^ The patient must also be informed about the possibility of recurrence of the disease on the treated area. A long-term follow-up study of patients treated with the non cultured epidermal cell suspension technique demonstrated that finger and toe involvement in patients treated in other regions and NSV are independent risk factors for recurrence.^102^

For the patient to have an accurate expectation, he/she must be well informed that repigmentation and color homogenization can take from two months to even more than a year to occur, and that surgery is a treatment and not a cure, as there is always the possibility of disease recurrence.^80^

There is evidence that the association of surgical transplantation techniques with adjuvant phototherapy provides better rates of repigmentation, which can be further improved when phototherapy is started before surgical treatment and continued after melanocyte transplantation.^103^, ^104^

### Other repigmentation-inducing techniques

The number of studies (trials and case reports) involving repigmentation-inducing techniques (RIT) without melanocyte transplantation is increasing, with no direct comparative studies between the two modalities to date.

Among RIT, microneedling stands out. The studies already published present a highly variable response rate (0–100%), many of them pointing to a lower response or even failure of response in acral areas, which can be explained by the lack of a follicular reservoir of melanocytes in these anatomical areas.^105^, ^106^, ^107^, ^108^, ^109^, ^110^, ^111^, ^112^, ^113^, ^114^, ^115^, ^116^, ^117^

Nonetheless, the technique is still considered incipient, albeit promising, similarly to the use of substances for drug delivery; therefore, it is prudent to await studies with a larger number of patients before its broad indication. It is important to note that, although the modality does not involve melanocyte transplantation, the same indication criteria adopted for the other surgical treatment modalities of vitiligo need to be respected, in order to avoid possible adverse effects.

## Treatment in children, pregnant women, nursing mothers, and the elderly

### Phototherapy

In the pediatric age group and in pregnant women, phototherapy with NB-UVB is preferable to PUVA therapy due to its greater effectiveness, lower incidence of side effects (no need for photosensitizing medication – risk category C in pregnancy), and lower chance of carcinogenesis.^118^ The decision to initiate phototherapy in children is based on little or no response to topical treatments, rapid progression of the disease, and the understanding of the patient to accept and collaborate with the treatment. This usually occurs at around 7–10 years of age. As far as possible, localized irradiation devices should be tried to avoid extensive and delayed damage.^119^

To date, studies with NB-UVB have not shown an increase in carcinogenesis in children with vitiligo, suggesting that its use may be safe from school age on.^120^, ^121^, ^122^, ^123^, ^124^, ^125^ Regarding vitiligo and carcinogenesis, most studies show an inverse relationship between the disease and skin cancer, probably due to protective genetic and immunological mechanisms.^126^ However, due to the absence of long-term prospective studies to certify the real safety of phototherapy in children, a long-term dermatological follow-up in treated patients and restriction of phototherapy to the treatment area, avoiding genital areas, is suggested.^121^, ^126^

It is also recommended that patients undergoing treatment or already treated reduce the environmental exposure to ultraviolet as much as possible and undergo regular examination of the entire tegument.^121^

As in adults, phototherapy combined with the topical use of calcineurin inhibitors can be indicated.^127^ However, the possible carcinogenic potential of this association must be taken into account. Although analyses of clinical data have not proven this possibility, combined use should be assessed with caution in children.

No specific studies in the elderly were retrieved.

### Oral corticosteroids

In children, the most common side effects of using oral corticosteroid therapy are vomiting, behavioral changes, and sleep disorders.^128^ The use of oral corticosteroids is also linked to an increased incidence of fractures both in the pediatric age group and in elderly patients.^129^, ^130^ Therefore, its use must be moderate and avoided in patients with moderate to high risk for fractures.

The literature review retrieved only one study of OMP prednisolone in children, a prospective and interventional study of 400 participants (children aged 18 months to 15 years), using two consecutive days of methylprednisolone for six months every week. In addition, patients used fluticasone cream once a day on the lesions. A response considered good to excellent was observed in 65.5% of the 343 patients who completed the study. There was an inverse correlation between disease duration and response to treatment.^131^

Although conflicting, some studies in pregnant women who used oral corticotherapy point to an increase in the incidence of cleft lip, preterm births, pre-eclampsia, and low birth weight, being considered a risk category C medication.^132^ Therefore, its use should be avoided in the pregnant population.

In the only retrospective study detailing the treatment of late-onset vitiligo (above 50 years of age), 15.6% of the 359 patients were treated with oral corticosteroids.^133^ That study cannot be extrapolated to the elderly age group, because many treated patients did not fit this age group.

### Topical treatment

The choice of topical medication in children should take into account the anatomical area of application and extent of the disease, and should be avoided to a great extent, due to the possibility of systemic side effects.^134^, ^135^ On the face, neck, and intertriginous areas, topical use of calcineurin inhibitors should be preferred (twice daily), due to their effectiveness and better safety profile.^134^, ^136^

According to the package insert, in Brazil, topical calcineurin inhibitors are indicated for children older than 2 years of age. However, these medications (tacrolimus 0.03% and pimecrolimus 1%) have already been studied in the age group of 6 to 24 months in patients with vitiligo, demonstrating efficacy and tolerability.^137^

Topical medium- and high-potency corticosteroids are the first line therapy for pediatric vitiligo on the body, except for intertriginous and genital sites. Children, in particular those with a high phototype and facial lesions, respond better than older individuals with lower phototypes. They are usually prescribed intermittently, with intervals of every two or three weeks, for a maximum of six months.^138^ A retrospective study that included 101 children with vitiligo treated with moderate to high potency steroids reported lesion repigmentation in 64% of the sample.

Due to its already known adverse local and systemic side effects, in the body region (extra-facial, extra-cervical, and extra-intertriginous) the recommendation is for the use of low- and moderate-potency corticosteroids, or newer high-potency corticosteroids (class III), such as mometasone furoate and methylprednisolone aceponate, which have similar efficacy and less common side effects than clobetasol propionate, for example.^134^

Systemic absorption is possible, especially in younger children, with the potential to suppress the hypothalamic-pituitary-adrenal axis and consequent iatrogenic Cushing's syndrome.^139^ High-potency topical corticosteroids produce better results in pediatric patients with head and neck lesions, but are not necessarily better than tacrolimus.^140^ Treatment plans that alternate periods of use with periods of pause (*e.g*., one week of use and one week of pause, or 15 days of treatment per month for six months) and that include monthly clinical follow-up to check for local side effects can be instituted. While in practice these treatment regimens reduce side effects, there are no comparative studies assessing their effectiveness.^134^, ^135^

The use of high and very high doses of topical corticosteroids during pregnancy has been linked to a higher incidence of low birth weight infants, especially in accumulated doses of 300 g or more.^141^ Therefore, a prudent use (reduced time and area of treatment) of topical corticosteroids of mild to moderate potency would be more indicated in this population, as well as in lactating women.^142^

There are no specific studies on the safety of using topical calcineurin inhibitors in pregnant and lactating women (Category C). However, the use of oral tacrolimus and even oral cyclosporine has already been studied in pregnant women who received solid organ transplants, showing no increased risk for congenital malformations. The risk of increased prematurity in these studies was possibly related to maternal-based disease.^143^, ^144^

Studies on the pharmacokinetics of topical tacrolimus in patients with atopic dermatitis (with greater permeability in the skin barrier than patients with vitiligo) have shown minimal systemic absorption of topical medication.^145^, ^146^ Therefore, the off-label use of this medication can be suggested in pregnant or lactating vitiligo patients, but as a second line of treatment.

Retrospective studies, including one carried out in a Brazilian population, indicate a trend toward the stability of vitiligo during pregnancy in the majority of patients evaluated.^147^, ^148^

In a retrospective study involving 359 patients with late onset of the disease (>50 years), only 30.5% of those treated with topical monotherapy (corticosteroids or calcineurin inhibitors) achieved good repigmentation results (repigmentation>50%), while the majority (55.4%) of those treated with phototherapy associated with topical medication achieved good to excellent therapeutic results.^149^ Thus, the present consensus recommends the latter combination therapy as a form of treatment for the elderly.

## Discussion and Conclusion

**Figure 1:**
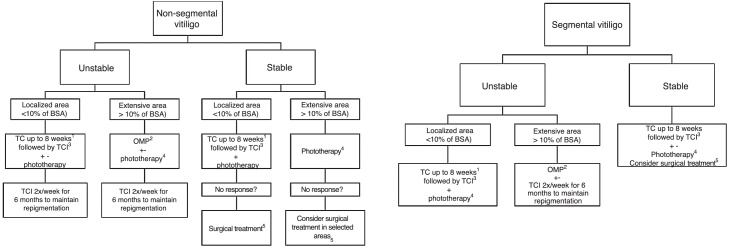
Flowchart of treatment. BSA, Body Surface Area; TC, Topical corticoid; TCI, Topical Calcineurin Inhibitor (Tacrolimus or Pimecrolimus); OMP, Oral Corticoid Mini-pulse. +-, Associated or not to. ^1^ With clinical monitoring to evaluate local side effects, especially if in genital, facial or skinfold areas. Most reviewers agree that, if the use of TC is necessary, preference should be given to those with low and medium potency in genital, skinfold and facial areas. The use of high and very-high potency corticosteroids should be restricted to the other body areas. Also, in sensitive areas, the use of TCI can be prioritized to minimize the side effects of topical corticosteroids. ^2^ In children and the elderly, evaluate the risk-benefit of using oral corticosteroid therapy, mainly due to the association between its use and growth deficit and increased fracture risk, in addition to comorbidities that can be triggered or aggravated by the medication use. ^3^ Tacrolimus (or Pimecrolimus) 2×/day. ^4^ Preferably NB-UVB, Excimer laser or Excimer light. Due to slow responders, a treatment lasting at least six months (2 to 3 weekly sessions) is suggested. In localized cases, give preference to phototherapy treatment that allows irradiation restricted to the lesion area. The association with oral anti-oxidants, TCI and TC during treatment can be considered. It was a consensus among the reviewers that the genital area should not be irradiated. ^5^ In the absence of Köbner phenomenon.

The consulted experts defined as the standard treatment of vitiligo the use of corticosteroids and calcineurin inhibitors for unstable and localized cases, and corticosteroid OMP for unstable generalized vitiligo. The treatment of choice for repigmentation is NB-UVB phototherapy, which may or may not be associated with OMP and/or oral antioxidants in severe and unstable cases. Surgical modalities are used mainly in stable SV and generalized vitiligo, primarily associated with exposure to phototherapy before and after surgical treatment. To date, there are no specific topical or systemic drugs for vitiligo, but the main drugs being developed are topical and systemic anti-JAKs, which are between Phase II and III; trials with anti-IL-15 receptor immunobiologicals are planned. Fig. 1 presents a treatment flowchart.

## Financial support

None declared.

## Authors’ contributions

Gerson Dellatorre: Approval of the final version of the manuscript; design and planning of the study; elaboration and writing of the manuscript; collection, analysis, and interpretation of data; effective participation in research orientation; critical review of the literature; critical review of the manuscript.

Daniela Alves Pereira Antelo: Approval of the final version of the manuscript; design and planning of the study; elaboration and writing of the manuscript, collection, analysis, and interpretation of data; effective participation in research orientation; critical review of the literature; critical review of the manuscript.

Roberta Buense Bedrikow: Approval of the final version of the manuscript; design and planning of the study; elaboration and writing of the manuscript; collection, analysis, and interpretation of data; effective participation in research orientation; critical review of the literature; critical review of the manuscript.

Tania Ferreira Cestari: Approval of the final version of the manuscript; design and planning of the study; elaboration and writing of the manuscript; collection, analysis, and interpretation of data; effective participation in research orientation; critical review of the literature; critical review of the manuscript.

Ivonise Follador: Approval of the final version of the manuscript; design and planning of the study; elaboration and writing of the manuscript; collection, analysis, and interpretation of data; effective participation in research orientation; critical review of the literature; critical review of the manuscript.

Daniel Gontijo Ramos: Approval of the final version of the manuscript; design and planning of the study; elaboration and writing of the manuscript; collection, analysis, and interpretation of data; effective participation in research orientation; critical review of the literature; critical review of the manuscript.

Caio Cesar Silva de Castro: Approval of the final version of the manuscript; design and planning of the study; elaboration and writing of the manuscript; collection, analysis, and interpretation of data; effective participation in research orientation; critical review of the literature; critical review of the manuscript.

## Conflicts of interest

None declared.
